# Efficacy of preoperative single-dose dexamethasone in preventing postoperative pulmonary complications following minimally invasive esophagectomy: a retrospective propensity score-matched study

**DOI:** 10.1186/s13741-024-00407-6

**Published:** 2024-05-28

**Authors:** Xiaoxi Li, Ling Yu, Jiaonan Yang, Miao Fu, Hongyu Tan

**Affiliations:** Key Laboratory of Carcinogenesis and Translational Research (Ministry of Education/Beijing), Department of Anesthesiology, Peking University Cancer Hospital & Institute, #52 Fucheng Street, Haidian District, Beijing, 100142 China

**Keywords:** Dexamethasone, Glucocorticoids, Minimally invasive esophagectomy, Postoperative pulmonary complications, Preoperative medication

## Abstract

**Background:**

The study was performed to investigate the efficacy and safety of preoperative dexamethasone (DXM) in preventing postoperative pulmonary complications (PPCs) after minimally invasive esophagectomy (MIE).

**Methods:**

Patients who underwent total MIE with two-field lymph node dissection from February 2018 to February 2023 were included in this study. Patients who were given either 5 mg or 10 mg DXM as preoperative prophylactic medication before induction of general anesthesia were assigned to the DXM group, while patients who did not receive DXM were assigned to the control group. Preoperative evaluations, intraoperative data, and occurrence of postoperative complications were analyzed. The primary outcome was the incidence of PPCs occurring by day 7 after surgery.

**Results:**

In total, 659 patients were included in the study; 453 patients received preoperative DXM, while 206 patients did not. Propensity score-matched analysis created a matched cohort of 366 patients, with 183 patients each in the DXM and control groups. A total of 24.6% of patients in the DXM group and 30.6% of patients in the control group had PPCs (*P* = 0.198). The incidence of respiratory failure was significantly lower in the DXM group than in the control group (1.1% vs 5.5%,* P* = 0.019). Fewer patients were re-intubated during their hospital stay in the DXM group than in the control group (1.1% vs 5.5%,* P* = 0.019).

**Conclusions:**

Preoperative DXM before induction of anesthesia did not reduce overall PPC development after MIE. Nevertheless, the occurrence of early respiratory failure and the incidence of re-intubation during hospitalization were decreased.

**Trial registration:**

Chinese Clinical Trial Registry (No. ChiCTR2300071674; Date of registration, 22/05/2023)

## Background

Major surgery is often associated with an increased inflammatory response, which may be related to the development of postoperative complications. Esophagectomy, one of the most invasive surgical procedures, is associated with high morbidity and mortality, especially with respect to pulmonary complications (Geller et al. 2019). A recent study showed that lung injury arising from the inflammatory response to surgical stress and oxidative stress is associated with pulmonary morbidity after esophagectomy (Shinozaki et al. 2021). To improve the postoperative clinical course, preoperative glucocorticoids have been introduced to attenuate the release of proinflammatory mediators such as IL-6, IL-8, and tumor necrosis factor (TNF) (Shinozaki et al. 2021; Takeda et al. 2003; Matsutani et al. 1998; Shimada et al. 2000). A few studies have investigated the protective effect of glucocorticoids in patients undergoing open esophagectomy (Gao et al. 2014; Nakamura et al. 2008). It is suggested that the benefits of cytokine response-modulating therapy may become blunted in the presence of major complications. Considering that minimally invasive esophagectomy (MIE) is associated with less severe surgical trauma and fewer postoperative complications, investigating the prophylactic effect of glucocorticoids on postoperative pulmonary complications (PPCs) following MIE might produce informative results.

The long-term glucocorticoid dexamethasone (DXM) is one of the most frequently used preanesthetic drugs because of its antiemetic and analgesic effects (Corcoran et al. 2022; Parthasarathy et al. 2018). In theory, the relatively long half-life of DXM may have a prolonged anti-inflammatory effect following surgery. However, whether prophylactic DXM before induction of anesthesia alleviates surgical stress and reduces postoperative complications (especially pulmonary complications) following MIE has never been exclusively studied. The aim of this study was to investigate the efficacy and safety of prophylactic DXM on PPC development after MIE.

## Methods

### Study population

All consecutive patients diagnosed with thoracic esophageal cancer who underwent total MIE with two-field lymph node dissection at a single academic institution (Peking University Cancer Hospital) from February 2018 to February 2023 were included in the study. Before the study’s commencement, approval was obtained from the hospital’s ethics committee (No. 2023YJZ36). The study was registered in the Chinese Clinical Trial Registry (No. ChiCTR2300071674) and was based on a previous retrospective study (Chinese Clinical Trial Registry, ChiCTR2300071571). The requirement for informed consent was waived because of the retrospective study design. In addition, patients who met one or more of the following criteria were excluded from the study: (1) esophageal cancer recurrence, (2) treatment with corticosteroids before surgery, (3) treatment with additional intraoperative corticosteroids except for prophylactic DXM, (4) American Society of Anesthesiologists (ASA) classification score greater than III, (5) COVID-19 infection within 1 month prior to surgery or during hospitalization, (6) change of operation method during surgery (e.g., unresectable tumor, conversion to an open procedure), and (7) incomplete data. All operations were performed by one of three senior esophageal cancer surgeons from the same ward, and general anesthesia was administered by the same team of thoracic anesthesiologists. Postoperative treatment was managed by clinicians on the same general ward or in the intensive care unit (ICU). All patients’ electronic medical records were reviewed for baseline characteristics, operative information, and postoperative variables during the hospital stay. Preoperative evaluation included a complete medical history, physical examination, and relevant laboratory tests and investigations. Pulmonary history was defined as a history of chronic obstructive pulmonary disease, bronchiectasis, asthma, pulmonary bullae, pulmonary infection within 1 month, and/or pulmonary tuberculosis. Intraoperative data included operative records and anesthesia records. Postoperative follow-up consisted of the duration of ICU stay/hospital stay, postoperative laboratory and imaging data, and the occurrence of PPCs and non-pulmonary complications.

### Anesthesia and surgical technique

All patients underwent general anesthesia with one-lung ventilation or two-lung ventilation under artificial pneumothorax. Epidural anesthesia was not routinely used for patients undergoing total MIE in our institution. Regional anesthesia such as paravertebral block, transversus abdominis plane block, and intercostal nerve block were applied at the attending anesthesiologists’ discretion. A glucocorticoid (5 mg or 10 mg DXM) was administered as a preoperative prophylactic medication before induction of general anesthesia at the anesthesiologists’ discretion. Intubation was completed with either a double-lumen endotracheal tube, a single-lumen endotracheal tube, or the combination of a single-lumen tube and a bronchial blocker after intravenous induction of general anesthesia. A protective lung ventilation strategy was adopted; i.e., a tidal volume of 4 to 6 mL/kg and a positive end-expiratory pressure of 2 to 5 cm H_2_O. General anesthesia was maintained with a combination of inhaled and intravenous anesthesia. Patient-controlled intravenous analgesia with opioids was used for postoperative analgesia for all patients. The MIE procedure included two-field lymph node dissection with anastomosis in the neck, which consisted of right transthoracic subtotal esophagectomy and dissection of mediastinal and abdominal lymph nodes. The thoracic procedures were performed by thoracoscopic surgery in the left lateral-prone position. The abdominal procedures were performed by total laparoscopic surgery. After surgery, all patients were transferred to the post-anesthesia care unit and then either back to the general ward after full recovery or to the ICU if continued mechanical ventilation was necessary or severe comorbidities were present.

### Variables and data extraction

Data on PPCs were collected during the hospital stay. The primary outcome was the incidence of PPCs occurring by postoperative day (POD) 7, assuming that PPCs related to intraoperative management would occur early. Late PPCs may be associated with factors other than the initial surgery, such as aspiration or wound infection (Howells et al. 2016). PPCs were defined as grade ≥ II complications according to the Clavien-Dindo classification of respiratory system complications (Table 1) (Dindo et al. 2004). Major pulmonary complications occurring by POD 7 were analyzed separately, including pneumonia (defined as the presence of a new or progressive radiographic infiltrate plus at least two of three clinical features including fever of > 38 °C, leukocytosis or leukopenia, and purulent secretions), pleural effusion requiring an additional drainage procedure, atelectasis requiring bronchoscopic treatment, pneumothorax requiring thoracocentesis, respiratory failure (defined as postoperative PaO_2_ of < 60 mmHg or arterial oxyhemoglobin saturation measured with pulse oximetry of < 90% despite oxygen therapy, or the need for non-invasive mechanical ventilation), acute respiratory distress syndrome, acute aspiration, and tracheobronchial injury. The secondary outcome was non-pulmonary complications occurring during the hospital stay, which included anastomotic leakage, wound infection, cardiac complications (including new-onset arrhythmia, myocardial ischemia or infarction, and heart failure), chylothorax, and recurrent laryngeal nerve injury. The length of the hospital stay was also analyzed.

**Table 1:** Pulmonary complication grading scheme adapted from the Clavien-Dindo classification (Dindo et al. 2004)

Grades	Definition	Examples of respiratory system
Grade I	Any deviation from the normal postoperative course without the need for pharmacological treatment or surgical, endoscopic, and radiological interventions Allowed therapeutic regimens are: drugs as antiemetics, antipyretics, diuretics, electrolytes, and physiotherapy. This grade also includes wound infections opened at the bedside	Secretion retention requiring physiotherapy Atelectasis requiring physiotherapy Therapeutic supplemental oxygen given by nasal catheter or oxygen mask
Grade II	Requiring pharmacological treatment with drugs other than such allowed for grade I complications Blood transfusions and total parenteral nutrition are also included	Pneumonia treated with antibiotics on the ward
Grade III	Requiring surgical, endoscopic, or radiological intervention	Suction of secretions by bronchoscopy for treatment of pneumonia Atelectasis requiring bronchoscopic intervention
Grade IIIa	Intervention not under general anesthesia	
Grade IIIb	Intervention under general anesthesia	
Grade IV	Life-threatening complication requiring IC/ICU management	Readmission to the ICU because of respiratory dysfunction
Grade IVa	Single organ dysfunction (including dialysis)	Respiratory failure requiring endotracheal or noninvasive ventilation
Grade IVb	Multiorgan dysfunction	Respiratory failure with failure of another organ
Grade V	Death of a patient	Death

*IC* intermediate care, *ICU* intensive care unit

### Statistical analysis

IBM SPSS Statistics for Windows, version 26.0 (IBM Corp., Armonk, NY, USA) was used for statistical analysis. Categorical variables are reported as numbers (percentage) and were analyzed with Pearson’s chi-squared test or Fisher’s exact test. Continuous variables were reported as mean ± standard deviation or median (interquartile range). Normally distributed data are analyzed with the independent-samples *t*-test, and non-normally distributed data are analyzed with the Mann–Whitney *U* test. To adjust for unbalanced covariates between the patients who received DXM preoperatively (DXM group) and patients who did not (control group), propensity score-matched analysis was used. Baseline characteristics and preoperative/intraoperative data were analyzed, including age, sex, height, weight, body mass index (BMI), ASA classification, comorbidities, hemoglobin concentration, albumin concentration, preoperative treatment, tumor location, tumor pathology, and anesthesia technique. Variables with a *P* value of < 0.1 in the univariate analysis between the two groups and perioperative risk factors reported to be associated with PPCs (e.g., age, BMI, pulmonary comorbidity, former smoking status, ventilation technique, duration of surgery, intraoperative fluid intake, and operative blood loss (Kinugasa et al. 2004; Zingg et al. 2011; Uchihara et al. 2018; Ohi et al. 2019; Law et al. 2004; Xing et al. 2015) were included for propensity calculation and matching using a logistic regression model. By setting the matching ratio at 1:1 using the nearest-neighbor method and the caliper width at 0.2 of the standard deviation, two adequately balanced groups (DXM group and control group) were generated for further analysis. A standardized mean difference of < 0.1 between the two groups was considered adequately balanced in matching. Statistical significance was set at *P* < 0.05.

## Results

**Fig. 1:**
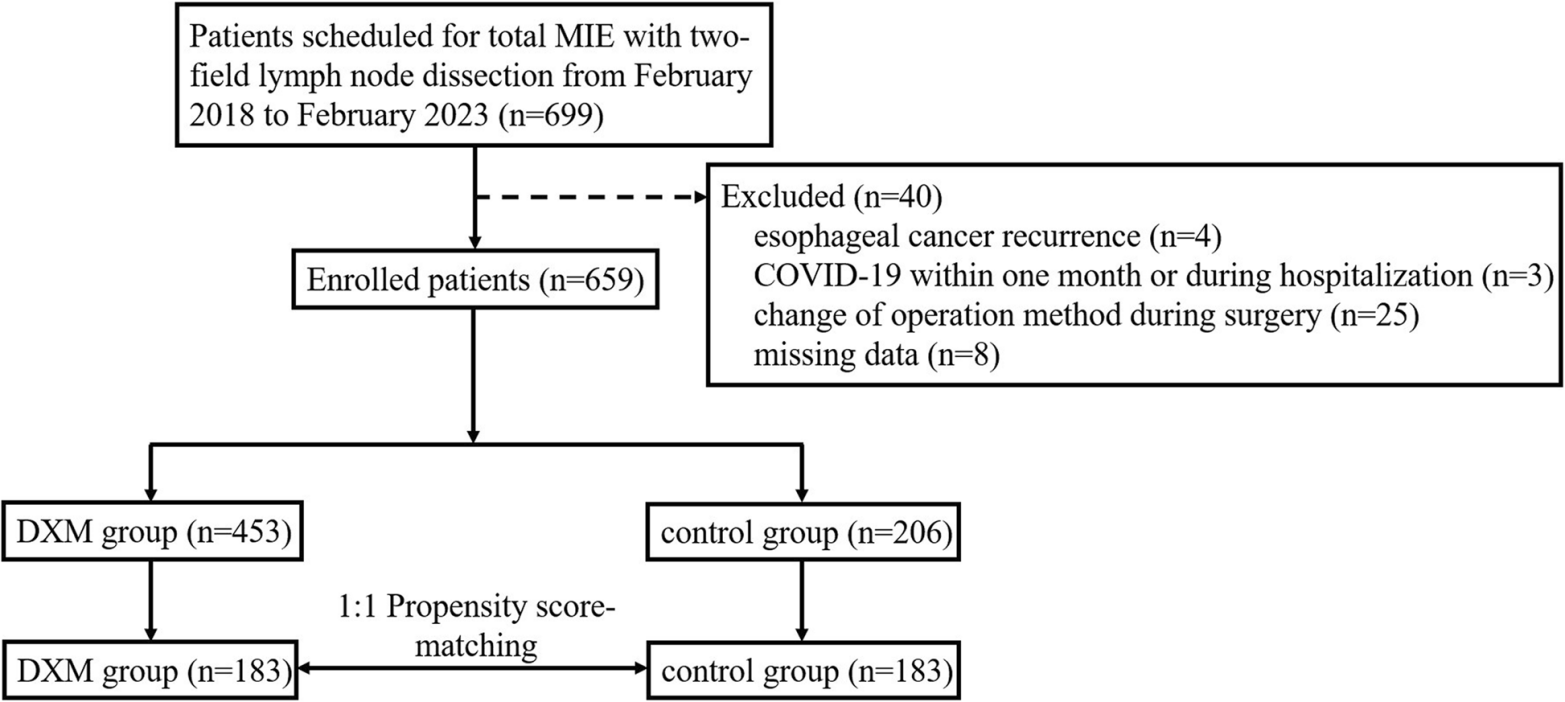
Flow chart of patient enrollment

During the study period, 699 patients were scheduled for total MIE with two-field lymph node dissection. A total of 659 patients qualified for enrollment and were included in the study. Among those individuals, 453 patients received prophylactic DXM and 206 patients did not. Prior to matching, there were significant variations in the current smoking status, ASA classification, tumor location, intraoperative fluid intake, and percentage of patients receiving nerve block between the two groups (Table 2). Therefore, the following risk factors were adjusted to compensate for the propensity score: age, BMI, pulmonary comorbidities, smoking status, ASA classification, tumor location, percentage of patients receiving combined nerve block, intraoperative fluid intake, operative blood loss, and duration of surgery. Propensity score-matching created a matched cohort of 366 patients with 183 patients each in the DXM group and the control group (Fig. 1). After matching, there were no significant differences in demographic characteristics or intraoperative data between the two groups (Table 2).

**Table 2:** Demographic and perioperative characteristics before and after propensity score-matching

Characteristics	Before matching (*n* = 659)			After matching (*n* = 366)		
	DXM group (*n* = 453)	Control group (*n* = 206)	*P* value	DXM group (*n* = 183)	Control group (*n *= 183)	*P* value
Age, years	64.0(59.0–69.0)	63.0(57.0–68.0)	0.292	63.0(58.0–68.0)	64.0(57.0–68.0)	0.849
Gender, male	370(81.7%)	175(85.0%)	0.303	146(79.8%)	153(83.6%)	0.344
Height, cm	166.0(161.0–172.0)	166.0(162.0–171.0)	0.810	166.0(161.0–170.0)	166.0(162.0–171.0)	0.609
Weight, kg	65.0(57.5–71.0)	66.0(58.0–71.0)	0.475	65.0(57.5–70.0)	65.0(57.5–71.0)	0.638
BMI, kg/m^2^	23.5(21.3–25.7)	23.6(22.0–25.7)	0.428	23.6 ± 3.0	23.6 ± 2.9	0.945
Pulmonary comorbidities	53(11.7%)	15(7.3%)	0.084	14(7.7%)	15(8.2%)	0.847
COPD	28(6.2%)	8(3.9%)	0.229	5(2.7%)	8(4.4%)	0.397
Pulmonary infection within a month	14(3.1%)	3(1.5%)	0.220	7(3.8%)	3(1.6%)	0.200
Diabetes mellitus	60(13.2%)	20(9.7%)	0.198	26(14.2%)	17(9.3%)	0.144
Smoking history						
Past smoker	316(69.8%)	139(67.5%)	0.557	124(67.8%)	123(67.2%)	0.911
Current smoker	23(5.1%)	19(9.2%)	0.043^*^	14(7.7%)	14(7.7%)	1.000
Alcohol abuse	237(52.3%)	112(54.4%)	0.625	89(48.6%)	101(55.2%)	0.209
Hemoglobin, g/L	129.7 ± 15.4	129.2 ± 17.0	0.673	128.4 ± 15.7	129.6 ± 16.8	0.487
Albumin, g/L	44.2(41.9–46.1)	44.2(41.4–46.0)	0.773	44.4(42.6–46.2)	44.4(41.7–46.4)	0.571
ASA classification			0.000^*^			0.493
I	12(2.6%)	25(12.1%)		11(6.0%)	17(9.3%)	
II	400(88.3%)	180(87.4%)		170(92.9%)	165(90.2%)	
III	41(9.1%)	1(0.5%)		2(1.1%)	1(0.5%)	
Neoadjuvant therapy			0.665			0.316
Yes	350(77.3%)	156(75.7%)		146(79.8%)	138(75.4%)	
No	103(22.7%)	50(24.3%)		37(20.2%)	45(24.6%)	
Tumor location			0.048^*^			0.940
Upper thoracic	62(13.7%)	18(8.7%)		19(10.4%)	17(9.3%)	
Middle thoracic	198(43.7%)	109(52.9%)		92(50.3%)	93(50.8%)	
Lower thoracic^a^	193(42.6%)	79(38.3%)		72(39.3%)	73(39.9%)	
Pathology			0.948			0.849
SCC	426(94.0%)	195(94.7%)		175(95.6%)	173(94.5%)	
Adenocarcinoma	20(4.4%)	8(3.9%)		7(3.8%)	8(4.4%)	
Other malignancy	7(1.5%)	3(1.5%)		1(0.5%)	2(1.1%)	
Ventilation technique			0.152			0.626
One-lung ventilation	72(15.9%)	24(11.7%)		20(10.9%)	23(12.6%)	
Two-lung ventilation	381(84.1%)	182(88.3%)		163(89.1%)	160(87.4%)	
Combined nerve block	41(9.1%)	0(0.0%)	0.000^*^	0(0.0%)	0(0.0%)	–
Fluid intake, mL	2100.0(1800.0–2525.0)	2350.0(2100.0–2600.0)	0.000^*^	2300.0(2000.0–2800.0)	2300.0(2100.0–2550.0)	0.811
Blood loss, mL	100.0(50.0–100.0)	100.0(50.0–100.0)	0.919	100.0(50.0–100.0)	100.0(50.0–100.0)	0.782
Intraoperative transfusion	39(8.6%)	11(5.3%)	0.142	16(8.7%)	9(4.9%)	0.147
Surgery duration, min	198.0(175.0–233.0)	201.0(178.0–234.0)	0.626	192.0(170.0–232.0)	201.0(178.0–234.0)	0.177

Data are presented as median (interquartile range), mean ± standard deviation, or *n* (%)

*DXM* dexamethasone, *BMI* body mass index, *COPD* chronic obstructive pulmonary disease, *ASA* American Society of Anesthesiologists, *SCC* squamous cell carcinoma

^*^P < 0.05

^a^Includes the gastroesophageal junction

**Fig. 2:**
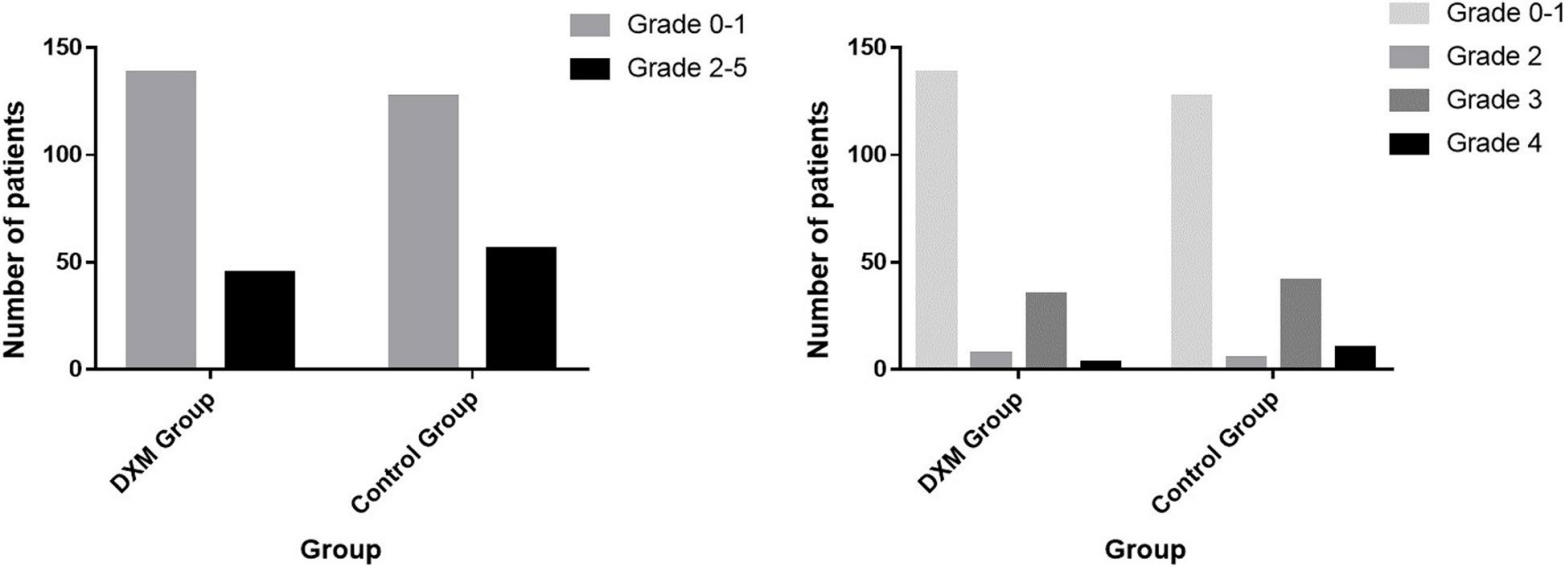
PPC grades by postoperative day 7 defined by the Clavien-Dindo classification

After matching, 45 (24.6%) patients in the DXM group and 56 (30.6%) patients in the control group had PPCs by POD 7 (*P* = 0.198) (Fig. 2). The incidence of respiratory failure by POD 7 was significantly lower in the DXM group than in the control group (1.1% vs 5.5%,* P* = 0.019). The incidence of other major PPCs by POD 7 did not differ significantly between the two groups (Table 3). Fewer patients were re-intubated during their hospital stay in the DXM group than in the control group [2 (1.1%) vs 10 (5.5%),* P* = 0.019].

**Table 3:** Occurrences of major PPCs requiring treatment by postoperative day 7

PPCs	DXM group (*n* = 183)	Control group (*n* = 183)	*χ*^2^	*P* value
Pneumonia	20(10.9%)	21(11.5%)	0.027	0.868
Pleural effusion	18(9.8%)	24(13.1%)	0.968	0.325
Pneumothorax	2(1.1%)	1(0.5%)	–	1.000
Atelectasis	5(2.7%)	5(2.7%)	0.000	1.000
Respiratory failure	2(1.1%)	10(5.5%)	5.514	0.019^*^
ARDS	0(0.0%)	1(0.5%)	0.000	1.000
Acute aspiration	0(0.0%)	0(0.0%)	–	–
Tracheobronchial injury	0(0.0%)	0(0.0%)	–	–
Re-intubation	2(1.1%)	8(4.4%)	3.701	0.054
Tracheotomy	2(1.1%)	5(2.7%)	1.311	0.252

Data are presented as *n* (%). *P < 0.05

*PPCs* postoperative pulmonary complications, *DXM* dexamethasone, *ARDS* acute respiratory distress syndrome

The incidence of postoperative cardiac complications was significantly lower in the DXM group (2.2%) than in the control group (6.6%, *P* = 0.041). The occurrences of other major postoperative non-pulmonary complications during the hospital stay were comparable between the two groups (Table 4). The length of hospital stay was 12.0 (10.0–14.0) days in the DXM group and 13.0 (11.0–15.0) days in the control group (*P* = 0.174).

**Table 4:** Occurrences of major postoperative non-pulmonary complications during hospital stay

Complications	DXM group (*n* = 183)	Control group (*n* = 183)	*χ*^2^	*P* value
Anastomotic leakage	18(9.8%)	17(9.3%)	0.032	0.859
Wound infection	6(3.3%)	3(1.6%)	0.456	0.500
Cardiac complications	4(2.2%)	12(6.6%)	4.183	0.041^*^
Chylothorax	1(0.5%)	1(0.5%)	0.000	1.000
Recurrent laryngeal injury	2(1.1%)	7(3.8%)	2.848	0.091

Data are presented as *n* (%)

*DXM* dexamethasone

^*^P < 0.05

## Discussion

In the present study, 24.6% of patients in the DXM group and 30.6% of patients in the control group developed PPCs by POD 7. There was a non-significant trend of reduced PPCs in patients treated with preoperative DXM administration. When major PPCs were analyzed separately, the incidence of respiratory failure was significantly lower in patients receiving preoperative DXM. This finding was consistent with several previous studies on other prophylactic glucocorticoids. Recently published Japanese nationwide inpatient data showed an association between prophylactic methylprednisolone use in oncologic esophagectomy and decreased respiratory failure as well as lower in-hospital mortality (Hirano et al. 2022). Likewise, an investigation into the effect of preoperative methylprednisolone on surgery-related complications following esophagectomy revealed that patients who received a prophylactic glucocorticoid had a significantly lower rate of organ system failure, including respiratory failure. In that investigation, patients in the glucocorticoid group had lower plasma levels of IL-6 and IL-8 than patients in the control group, indicating that glucocorticoids might have attenuated surgical stress–induced inflammatory responses by protecting against elevation of proinflammatory cytokine levels (Sato et al. 2002). In particular, the authors suggested that the improvement of respiratory function was related to the shorter duration of mechanical ventilation in patients who received prophylactic glucocorticoids. In the present study, the duration of mechanical ventilation was not analyzed; however, the re-intubation rate within POD 7 was lower in the DXM group (although the difference was not statistically significant), and the overall re-intubation rate during hospitalization was significantly lower in patients who received preoperative DXM. This may have resulted in a shorter duration of mechanical ventilation. Another investigation also revealed that intraoperative methylprednisolone administration was associated with a decreased risk of acute respiratory failure following esophagectomy (Park et al. 2012). Consistent results were reported in other studies, in which methylprednisolone pretreatment was associated with reduced intubation days, a higher postoperative ratio of arterial oxygen partial pressure to fractional inspired oxygen, and fewer postoperative respiratory complications (Takeda et al. 2003; Raimondi et al. 2006; Tsukada et al. 2006). These findings suggest a potential benefit of preoperative corticosteroid use in both open esophagectomy and MIE. On the basis of previous research and the results of the present study, we consider preoperative DXM effective in reducing the occurrence of respiratory failure following MIE. The mechanism underlying this association is unclear. The inflammatory response triggered by major surgery may induce proinflammatory cytokine secretions, endothelial dysfunction, glycocalyx damage, and neutrophil activations, leading to tissue and multisystem organ destruction (Margraf et al. 2020). It is suggested that lung injury induced by the inflammatory response to surgical stress and oxidative stress is associated with pulmonary morbidity after esophagectomy (Shinozaki et al. 2021). Previous studies have shown that glucocorticoids may lower the risk of respiratory failure by inhibiting the inflammatory response to surgical stress through suppression of proinflammatory cytokine release (Takeda et al. 2003; Sato et al. 2002; Raimondi et al. 2006). In one study, for example, the plasma norepinephrine and arginine vasopressin concentrations were significantly lower in patients treated with methylprednisolone than in the control group (Takeda et al. 2003). The postoperative plasma IL-6 concentration was also found to be lowered by preoperative glucocorticoid administration (Raimondi et al. 2006). The laboratory data in another study suggested that corticosteroids may attenuate surgical stress-induced inflammatory responses both directly by suppressing the release of proinflammatory cytokines and by inducing IL-10 synthesis (Sato et al. 2002). However, because of the retrospective design of the present study, data reflecting inflammatory responses could not be collected. Further studies are required to better understand the possible mechanism associated with the protective effect of respiratory function by preoperative DXM.

In addition to a reduced incidence of respiratory failure, we observed fewer postoperative cardiac complications in the DXM group. These findings were similar to previous studies, in which reduced cardiovascular failure or reduced cardiovascular complications were observed in patients treated with glucocorticoids (Gao et al. 2014; Sato et al. 2002; Engelman and Maeyens 2010). Generally, a single dose of glucocorticoid can inhibit the synthesis of proinflammatory mediators such as IL-6, IL-8, and TNF-α in cardiac surgery and other major non-cardiac surgeries such as procedures involving the esophagus and abdomen (Matsutani et al. 1998; Yilmaz et al. 1999; Schulze et al. 1997). Research has shown that higher levels of circulatory cytokines such as IL-6, IL-8, and TNF-α are closely related to impaired hemodynamics and may contribute to postoperative myocardial ischemia and deteriorated clinical outcomes after cardiac surgery (Deng et al. 1996; Hennein et al. 1994). Notably, a single dose of glucocorticoid was associated with a lower incidence of postoperative atrial fibrillation and fewer cases of myocardial injury after cardiac surgery (Liu et al. 2021; Xu et al. 2011; Zhou et al. 2023). However, inconsistent conclusions have been drawn regarding the influence of preoperative glucocorticoids on postoperative cardiac complications (Chaney et al. 2001). According to our results, we speculate that there may be a link between a reduced rate of postoperative cardiac complications and preoperative DXM administration. However, this speculation must be verified by future investigations.

The possible association between glucocorticoid use and surgical site complications such as impaired wound healing is a major concern (Wang et al. 2013; Bootsma et al. 2018). In one study, the incidence of graft dehiscence was higher in patients who received preventive hydrocortisone than in the control group (Jeong et al. 2019). In another study investigating postoperative acute lung injury after esophagectomy, superimposed infections were observed in 30% of the patients treated with corticosteroids, and surgical site complications (including wound dehiscence and anastomotic site leakage) were observed in 27% of the patients (Choi et al. 2019). This indicates the need for special attention to surgical complications related to glucocorticoids. Although the type and dosage of corticosteroids used in this study were not clarified, we speculate that a higher dosage was used because corticosteroids were used to treat acute respiratory distress syndrome. By contrast, whereas several research groups reported no adverse influence of glucocorticoid administration on anastomotic leakage, severe infection, and suture failure, two studies revealed the opposite results, showing protective effects of methylprednisolone pretreatment on surgical anastomotic leakage (Gao et al. 2014; Hirano et al. 2022; Park et al. 2012; Engelman and Maeyens 2010; Weijs et al. 2014). An investigation including a large sample of 8725 participants undergoing non-urgent, non-cardiac surgery demonstrated that 8 mg of intravenous DXM was non-inferior to placebo with respect to the incidence of surgical site infection (Corcoran et al. 2021). Similar to the dosage administered in that study, 5 mg or 10 mg of prophylactic DXM was used in the present study, and comparable occurrences of postoperative complications of anastomotic leakage and wound infection between the two groups were observed. These results suggest that a low dose of DXM pretreatment may not increase the incidence of adverse effects following MIE. However, because of the lack of consensus evidence produced by previous studies, further large-scale studies on the potentially negative impact of prophylactic DXM following MIE are warranted.

The clinical benefits and risks associated with preoperative glucocorticoid use remain unclear because of conflicting study results and lack of thorough investigation (Jeong et al. 2019; Choi et al. 2019; Yano et al. 2005). To our knowledge, although a few studies have investigated the effect of perioperative glucocorticoids on esophagectomy, most of the trials included a small number of participants and are now outdated (Takeda et al. 2003; Matsutani et al. 1998; Sato et al. 2002; Takeda et al. 1997). Studies focusing on the impact of prophylactic glucocorticoids on PPCs following MIE are also limited. The present study is the first to focus exclusively on the impact of preoperative DXM on the development of PPCs following MIE. The strengths of our study are our analysis of recent data (within 5 years) reflecting current practice and our focus on PPC development in patients undergoing MIE. Additionally, although previous studies have investigated hydrocortisone and methylprednisolone, there has been no thorough investigation on prophylactic DXM despite the fact that this glucocorticoid is one of the most frequently used pre-medications before anesthetic induction. Our study fills this gap.

This study has several limitations. First, this was a retrospective study in a single center, and the results may have therefore been affected by biases such as variability in standard practices among clinicians. However, all participants underwent propensity score-matched analysis to ensure maximal uniformity in patient characteristics as well as preoperative and intraoperative variables, including anesthesia-related factors; this analysis may have overcome some bias. Second, the anti-inflammatory effects of glucocorticoids were suggested to be dose-dependent. Nevertheless, the DXM group in the present study received a fixed dose of either 5 mg or 10 mg of DXM; the effects under different dosages were not studied separately. Thus, the optimum dosage of prophylactic DXM requires further investigation. Third, although preoperative corticosteroids may have an impact on the perioperative blood glucose concentration, these data were missing for some of the patients because of the retrospective study design and were therefore not included in the analysis. However, we collected data on postoperative wound infection, which is the most concerning postoperative complication in patients with perioperative hyperglycemia, and these data did not differ significantly between the two groups. Fourth, the postoperative pain score and the occurrence of postoperative nausea and vomiting might have influenced our results; however, these data could not be collected because of the retrospective design. Finally, the results of secondary outcomes regarding postoperative non-pulmonary complications should be interpreted with caution; they might be biased because the propensity score-matched analyses were mainly based on variables associated with PPCs. Future investigations using large sample sizes and a prospective design are required for more information, especially regarding the underlying mechanism of the protective effect of preoperative DXM on PPCs.

## Conclusion

Preoperative DXM before induction of anesthesia did not reduce overall PPC development. However, the postoperative occurrence of respiratory failure and the incidence of re-intubation during hospitalization were decreased. The optimal dosage of prophylactic DXM should be thoroughly analyzed using prospective randomized controlled trials.

## Data Availability

The datasets used and analyzed during the current study are available from the corresponding author upon reasonable request.

## Abbreviations

IL: Interleukin
TNF: Tumor necrosis factor
MIE: Minimally invasive esophagectomy
PPCs: Postoperative pulmonary complications
DXM: Dexamethasone
ASA: American Society of Anesthesiologists
ICU: Intensive care unit
POD: Postoperative day
BMI: Body mass index
